# Factors associated with development of re-nonunion after primary revision in femoral shaft nonunion subsequent to failed intramedullary nailing

**DOI:** 10.1186/s13018-018-0886-y

**Published:** 2018-07-20

**Authors:** Jiang-ying Ru, Li-xin Chen, Fang-yong Hu, Dai Shi, Rao Xu, Jian-wei Du, Yun-fei Niu

**Affiliations:** 1 Department of Orthopedics, the Affiliated Hospital of Yangzhou University, Yangzhou University, Yangzhou, 225009 China; 2 Department of Emergency, Jiangsu Provincial Corps Hospital of the Chinese People’s Armed Police Force, Yangzhou, 225003 China; 3 Department of Orthopedics, Changhai Hospital Affiliated to Second Military Medical University, Shanghai, 200433 China

**Keywords:** Femoral shaft fracture, Intramedullary nailing, Nonunion, Primary revision, Re-nonunion

## Abstract

**Background:**

Currently, there remains a lack of consensus regarding factors predictive of complication such as re-nonunion after primary revision in femoral shaft nonunion subsequent to failed intramedullary nailing (IMN). A better understanding of prognostic factors could potentially reduce the risk of re-nonunion happening and allow patients to maximize their recovery in the most expeditious manner. Our study aims to identify risk factors in the development of re-nonunion after primary revision inclusive of exchanging reamed nailing (ERN) and augmentative compression plating (ACP) with IMN in situ for femoral shaft nonunion subsequent to failed IMN.

**Methods:**

A retrospective study was performed for 63 cases (61 patients) of femoral shaft nonunion subsequent to failed IMN, who were made primary revision with either ERN or ACP from June 2007 to June 2015. The following set of variables was selected based on the speculation that they would contribute to the outcome: sex (male or female), age, body mass index(BMI), smoking, alcohol abuse, cause of injury, fracture type, type of IMN (antegrade or retrograde), use of IMN locking screws(dynamic or static), site of nonunion, primary nonunion time, pathological type of nonunion, bone defect (mm), primary revision method (ERN or ACP), and adjuvant autogenous bone grafting (ABG) (yes or no). Univariate analysis and multiple regression were used to identify risk factors in the development of re-nonunion after primary revision with either ERN or ACP for femoral shaft nonunion subsequent to failed IMN. The minimum follow-up time was 1.5 years (standard deviation [SD] = 1.2, range 1.5–8 years).

**Results:**

Of 63 cases (61 patients) of femoral shaft nonunion subsequent to failed IMN, primary revision with ERN was performed in 33 (52.4%) cases and primary revision with ACP was performed in 30 (47.6%) cases. Adjuvant ABG procedure was undertaken in 39 (61.9%) cases during primary revisions. Re-nonunion was diagnosed as in 18 (28.6%) cases after primary revision with either ERN or ACP. There was a significant difference in time to union between patients treated with primary ERN and those with primary ACP (log-rank, *p* = 0.006). Furthermore, the difference was also statistically significant between patients with adjuvant ABG procedure and those without it (log-rank, *p* = 0.009). The relative risk factors included smoking, BMI, site of nonunion, bone defect, primary revision method, and adjuvant ABG procedure. However, primary revision method and adjuvant ABG procedure were shown to be two independent risk factors in multiple logistic regression analysis.

**Conclusions:**

Patients with excessive tobacco use, BMI ≥ 30 kg/m^2^, bone defect ≥ 5 mm, primary revision with ERN, and no adjuvant ABG procedure had a higher likelihood of developing re-nonunion. Of these risk factors, primary revision with ERN and no adjuvant ABG procedure were two strongest risk factors.

## Background

Intramedullary nailing (IMN) is the “gold standard” of treatment for adult femoral shaft fractures because only a short hospital stay is required and it allows an early active range of motion and weight-bearing [1]. The nonunion rate after failed IMN has been known to be low, ranging from 0.8 to 2% [2–5]. However, with the growing patients with femoral shaft fracture and widespread application of IMN technique, the nonunions seem to be more common than predicted, with a range of 6.3–12.5% [6, 7]. Despite the continuous advances of surgical solutions, there is little evidence for the optimal treatment due to diversity of situations. More unfortunately, it has been reported that there is still a re-nonunion rate of 4–27% after primary revision for femoral shaft nonunion subsequent to failed IMN [8].

Re-nonunion is a great functional and economical challenge for patients, as well as a treatment dilemma for orthopedic surgeons. Currently, there remains a lack of consensus regarding predictive factors in the development of re-nonunion. Maybe identification of prognostic factors is helpful for orthopedic surgeons to decide how to minimize pain and disability of patients by promoting osseous union. Therefore, it is crucial to determine both modifiable and non-modifiable prognostic factors for re-nonunion and adjust practice accordingly. The purpose of this study is to critically evaluate our therapeutic protocol and to identify principal risk factors in the development of re-nonunion after primary revision with either exchanging reamed nailing (ERN) or augmentative compression plating (ACP) for femoral shaft nonunion subsequent to failed IMN.

## Methods

Between June 2007 and June 2015, 168 patients with femoral nonunions were treated by surgery successively in our Department of Trauma Surgery, the Affiliated Hospital of Yangzhou University. Sixty-nine cases (67 patients) with femoral shaft nonunion after failed IMN underwent primary revisions inclusive of ERN and ACP. Patients were identified from the logbook in operation room, and all cases of records were retrieved.

The study protocol was conducted following good clinical practice guidelines. The Department of Trauma Surgery, the Affiliated Hospital of Yangzhou University, is a designated trauma referral center in Jiangsu Province of China. We defined primary fracture nonunion as no radiological or clinical signs of union after 6 months following high-energy injuries (falls > 2 m high, road traffic, and industrial accidents) or after 12 to 16 weeks following low-energy fractures(falls< 2 m high, sporting injuries), with no evidence of progression in the previous 3 months. Of the identified patients, all data were retrieved from the hospital electronic patient file system and included in the study’s database.

Inclusion criteria included patients aged 18–66 years old, skeletal maturity, femoral shaft nonunion after failed IMN revised primarily with either ERN or ACP, the integrity of radiographic data, and follow-up more than 1 year.

Exclusion criteria consisted of skeletal immaturity, septic nonunions, the presence of metaphysial or pathological fractures, recent administration of corticosteroid and immunosuppressive drugs, and patients combined with severe systemic diseases or lost in follow-up or died.

**Fig. 1:**
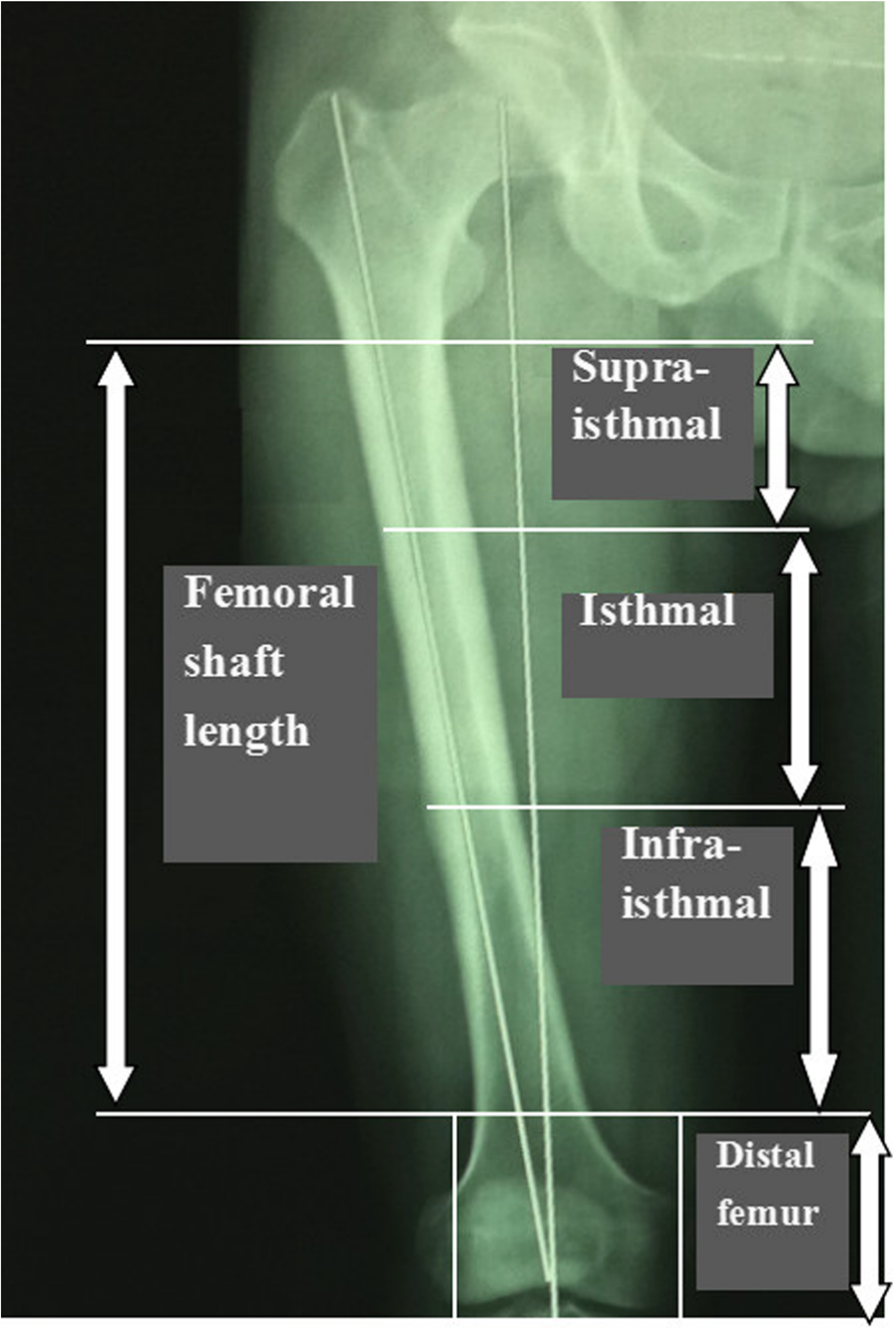
Definition of the length of the femoral shaft and its anatomic division into isthmal and non-isthmal (supraisthmal and infraisthmal) according to the metaphysial flare and isthmus

Patient demographics were recorded including age, sex, smoking, body mass index (BMI), alcohol abuse, cause of injury (traffic accidents, falling, or weight crushing), fracture type (open or closed), type of IMN (antegrade or retrograde), use of IMN locking screws (dynamic or static), site of nonunion (isthmal or non-isthmal), primary nonunion time, pathological type of nonunion (atrophic or hypertrophic), bone defect (mm), primary revision method (ERN or ACP), and adjuvant autogenous bone grafting (ABG). BMI cutoff value was identified as 30 kg/m^2^. Smoking was included as a host factor if the patient had a habit of ten or more cigarettes per day while alcohol abuse was identified if the reported weekly consumption exceeded 28 units for men or 14 units for women. The femoral shaft (diaphysis) level was defined, according to the Orthopaedic Trauma Association (OTA) classification, as the transverse lower edge of the lesser trochanter to the upper border of the transepicondylar width of the knee. Considering the isthmus and metaphysial flare, the nonunion site of the femoral shaft was divided into isthmal and non-isthmal (supraisthmal and infraisthmal) (Fig. 1) [9]. The definition and classification of nonunions were based on the Weber-Cech classification [10]. A bone defect at the site of nonunion was recorded if there was a distraction gap of 5 mm or bone loss of more than 50% of the femoral circumference at the nonunion site on plain radiographs. All of the risk factors that were present in each case were included in the statistical analysis.

All patients had obtained the continued follow-up until bone union completed, with the follow-up period more than 12 months. The results were retrospectively reviewed using the patients’ hospital and surgical charts. The clinical record and imaging data were independently reviewed by three of the authors (Ru JY, Chen LX, Hu FY).

ERN was performed on an ordinary radiolucent table under fluoroscopic guidance. After the original nail and cross-screws were removed through the original surgical incisions, intramedullary canal was initially reamed with a drill whose diameter was the same size as the extracted nail and overreamed several sizes with a 0.5-mm increment in reaming diameter sequentially. Reaming was undertaken until there was a “bony chatter” or healthy bone was seen on the tip of the withdrawn reamer. The increase in nail diameter ranged from 2 to 3 mm, with the average initial nail diameters at 11 mm (range 9–13 mm) and ERN (Synthes, Poli, PA) diameters at 13 mm (range 11–14 mm) respectively. Commonly static interlocking was applied. However, when having less risk of rotational instability or excessive shortening, dynamic interlocking may be performed. In addition, ABG from iliac crest was packed into the nonunion site which was exposed with minimal dissection of the periosteum from the surrounding healthy bone, if an obvious bone defect had been shown on radiographic data preoperatively.

ACP with adjuvant ABG procedure, regardless of pathological type of nonunions, was performed after exposing the nonunion site using the lateral approach with minimal soft tissue dissection. By rotating the distal femur, rotational instability was checked with the knee in a flexed position. Multiple drill holes and decortication of multiple small areas of the cortices near the fracture were carried out to increase raw bone surfaces. The fracture site was packed circumferentially with cortico-cancellous bone graft chips taken from the ipsilateral ilium wing. If distraction was present at the non-union site, removing interlocking bolts at one end of the IMN was needed to achieve compression. A 4.5-mm broad dynamic compression plate (Synthes, Poli, PA) or locking compression plate (Synthes, Poli, PA) was augmentatively placed in the lateral position of nonunion sites. The length of the plate was chosen to enable 3–4 screws placed in the proximal and distal fracture end respectively. Whether screws were placed either in front or behind the nail depended on anatomy of the femur, nail position, and direction needed to displace the fracture end either anterior or posterior in order to improve the reduction.

Bicortical screw purchase was routinely obtained in the metaphysis, but unicortical purchase was sometimes performed in the diaphysis.

All patients were encouraged to undertake mobilization under the supervision of a physiotherapist post-operatively. Patients treated with ERN were permitted to bear full weight within pain limits without a supportive cast or crutches immediately.

For assisting with mobility, elbow crutches were allowed to be used in the early post-operative period. However, patients treated with ACP were kept non-weight-bearing until there was radiographic evidence of incorporation of grafting bone and then advanced to full-weight-bearing based on clinical and radiographic evidence of healing. Patients were followed-up at 1, 2, 3, 4, 6, and 12 months after surgery, and then once every year. Radiographic confirmation of union at 1-month intervals need to be returned if there was no evidence of progression to union by 4 months.

Such outcomes needed to be measured as radiographic fracture union, failure of primary revisions to achieve union, and time to union etc. Fracture healing was radiographically as three solid bridging callus ridges connecting the fracture fragment on both anteroposterior (AP) and lateral views. Failure of primary revision with either ERN or ACP was defined as the requirement for further surgical intervention to achieve union. Time to union was identified as the time, in months, from primary revision with either ERN or ACP to radiographic union as defined above.

Categorical data were described using observed frequencies and percentages, and continuous variables were summarized by their means and standard deviations (or medians and interquartile ranges in case of serious deviations from normality).

The primary outcome was the occurrence of re-nonunion. The following set of predictive variables was selected based on our speculation that would contribute: sex, age, BMI, smoking, alcohol abuse, cause of injury, fracture type, type of IMN (antegrade or retrograde), use of IMN locking screws (dynamic or static), site of nonunion, primary nonunion time, pathological type of nonunion, bone defect (mm), primary revision method (ERN or ACP), and adjuvant ABG procedure. The univariate association of each predictor with outcome was assessed using a generalized estimating equation (GEE) logistic regression. In addition, a multivariable GEE logistic regression was performed that included all of the above variables. The probabilities of union and associated comparisons were estimated using the Kaplan-Meier test and log-rank analysis. All analyses were performed with SAS software (version 9.3; SAS Institute, Cary, NC, USA) by L-Biostat University of Leuven. All tests were two-sided and assessed at a significance level of 5%.

## Results

**Fig. 2:**
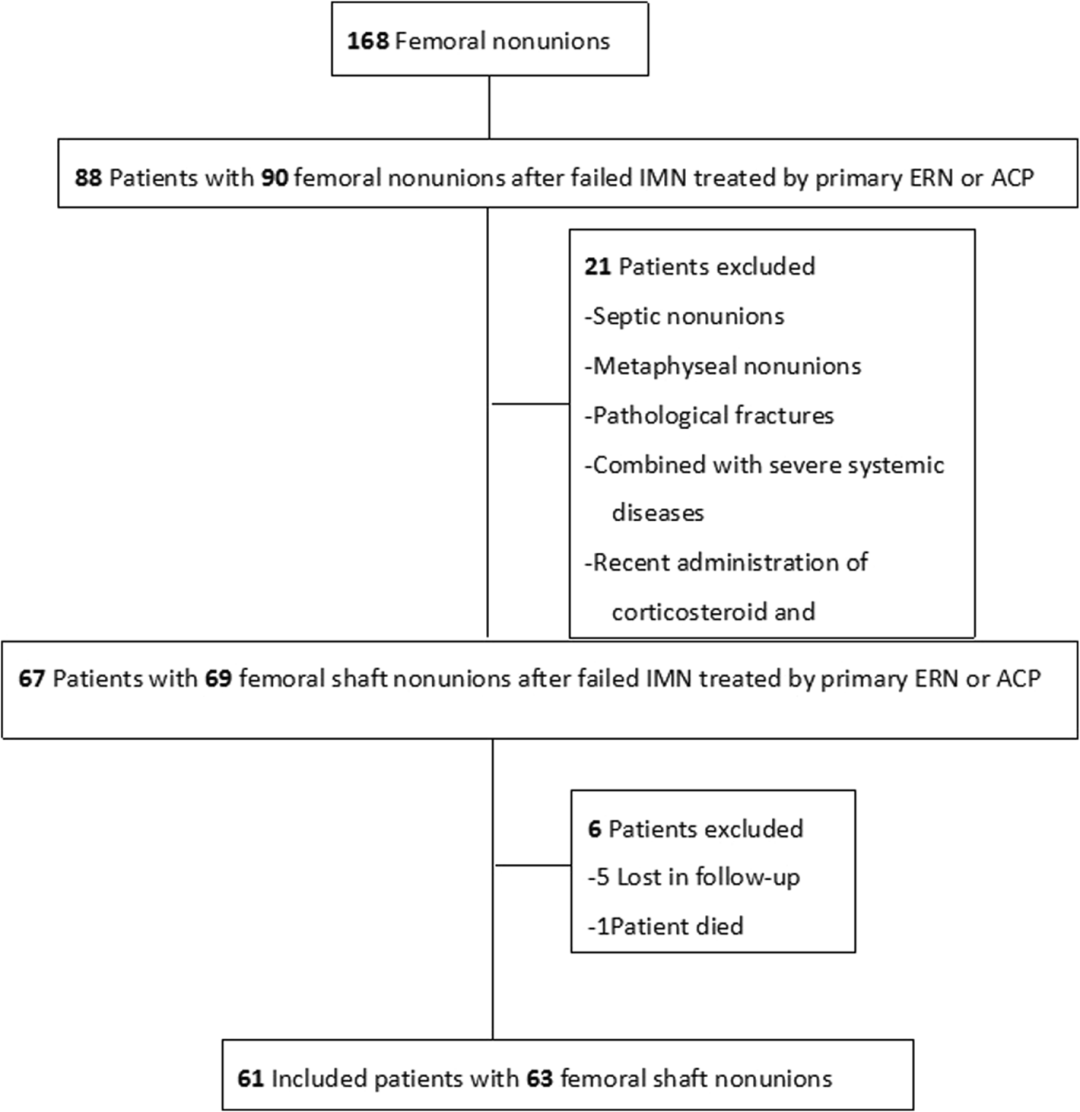
Flow diagram showing patient enrolment. IMN, intramedullary nailing; ERN, exchanging reamed nailing; ACP, augmentative compression plating

During the 8-year study period, 67 patients (69 cases) of femoral shaft nonunions after failed IMN conformed to the inclusion criteria. Of these, 61 patients (63 cases) obtained the follow-up, at a mean of 1.5 years (standard deviation[SD] = 1.2, range 1.5–8 years), and the mean age was 41.6 years (standard deviation[SD] = 14.0, range 18–66 years). The remaining five patients were lost to follow-up, and one died from operation-related causes within the first 30 days after primary revision surgery (Fig. 2). In this retrospective study, superficial wound infection happened in three patients who were all successfully treated with short-term (< 10 days) antibiotic treatment. There were no deep infections. Overall, 18 (28.6%) patients presented with re-nonunion, of whom 16 (88.9%) patients were revised primarily with ERN. The specific characteristic was summarized in Tables 1 and 2 with respect to the re-nonunion rate for each independent variable.

**Table 1:** Patient characteristics

Patients characteristics	Number (%)	Re-nonunion group 18 (29.5%)	Non-renonunion group 43 (70.5%)
Age (years)			
< 25	*N* (%)	4 (22.2%)	10 (23.3%)
25~ 45	*N* (%)	8 (44.4%)	21 (48.8%)
> 45	*N* (%)	6 (33.3%)	12 (27.9%)
Gender			
Male	*N* (%)	10 (55.6%)	23 (53.5%)
Female	*N* (%)	8 (44.4%)	20 (46.5%)
Smoking			
No	*N* (%)	5 (27.8%)	33 (76.7%)
Yes	*N* (%)	13 (72.2%)	10 (23.3%)
Alcohol abuse			
No	*N* (%)	7 (38.9%)	16 (37.2%)
Yes	*N* (%)	11 (61.1%)	27 (62.8%)
BMI (kg/m^2^)			
< 30	*N* (%)	6 (33.3%)	31 (72.1%)
≥ 30	*N* (%)	12 (66.7%)	12 (27.9%)
Cause of injury			
Traffic accidents	*N* (%)	8 (44.4%)	18 (41.9%)
Falling	*N* (%)	7 (38.9%)	14 (32.6%)
Weight crushing	*N* (%)	3 (16.7%)	11 (25.6%)

*N* total number of patients, *BMI* body mass index

**Table 2:** Nonunion characteristics

Nonunion characteristics	Number (%)	Re-nonunion group 18 (28.6%)	Non-renonunion group 45 (71.4%)
Fracture type			
Closed	*N* (%)	10 (55.6%)	24 (53.3%)
Open	*N* (%)	8 (44.4%)	21 (46.7%)
Type of IMN			
Antegrade	*N* (%)	11 (61.1%)	26 (57.8%)
Retrograde	*N* (%)	7 (38.9%)	19 (42.2%)
Use of IMN locking screws			
Dynamic	*N* (%)	6 (33.3%)	17 (37.8%)
Static	*N* (%)	12 (66.7%)	28 (62.2%)
Site of nonunion			
Isthmal	*N* (%)	5 (27.8%)	29 (64.4%)
Non-isthmal	*N* (%)	13 (72.2%)	16 (35.6%)
Primary nonunion time (years)			
< 1.5	*N* (%)	10 (55.6%)	25 (55.6%)
≥ 1.5	*N* (%)	8 (44.4%)	20 (44.4%)
Pathological type of nonunion			
Atrophic	*N* (%)	6 (33.3%)	15 (33.3%)
Hypertrophic	*N* (%)	12 (66.7%)	30 (66.7%)
Bone defect (mm)			
< 5	*N* (%)	7 (38.9%)	28 (62.2%)
≥ 5	*N* (%)	11 (61.1%)	17 (37.8%)
Primary revision method			
ERN	*N* (%)	16 (88.9%)	17 (37.8%)
ACP	*N* (%)	2 (11.1%)	28 (62.2%)
Adjuvant ABG procedure			
No	*N* (%)	13 (72.2%)	11 (24.4%)
Yes	*N* (%)	5 (27.8%)	34 (75.6%)

*N* total number of nonunions, *IMN* intramedullary nailing, *ERN* exchanging reamed nailing, *ACP* augmentative compression plating, *ABG* autogenous bone grafting

**Fig. 3:**
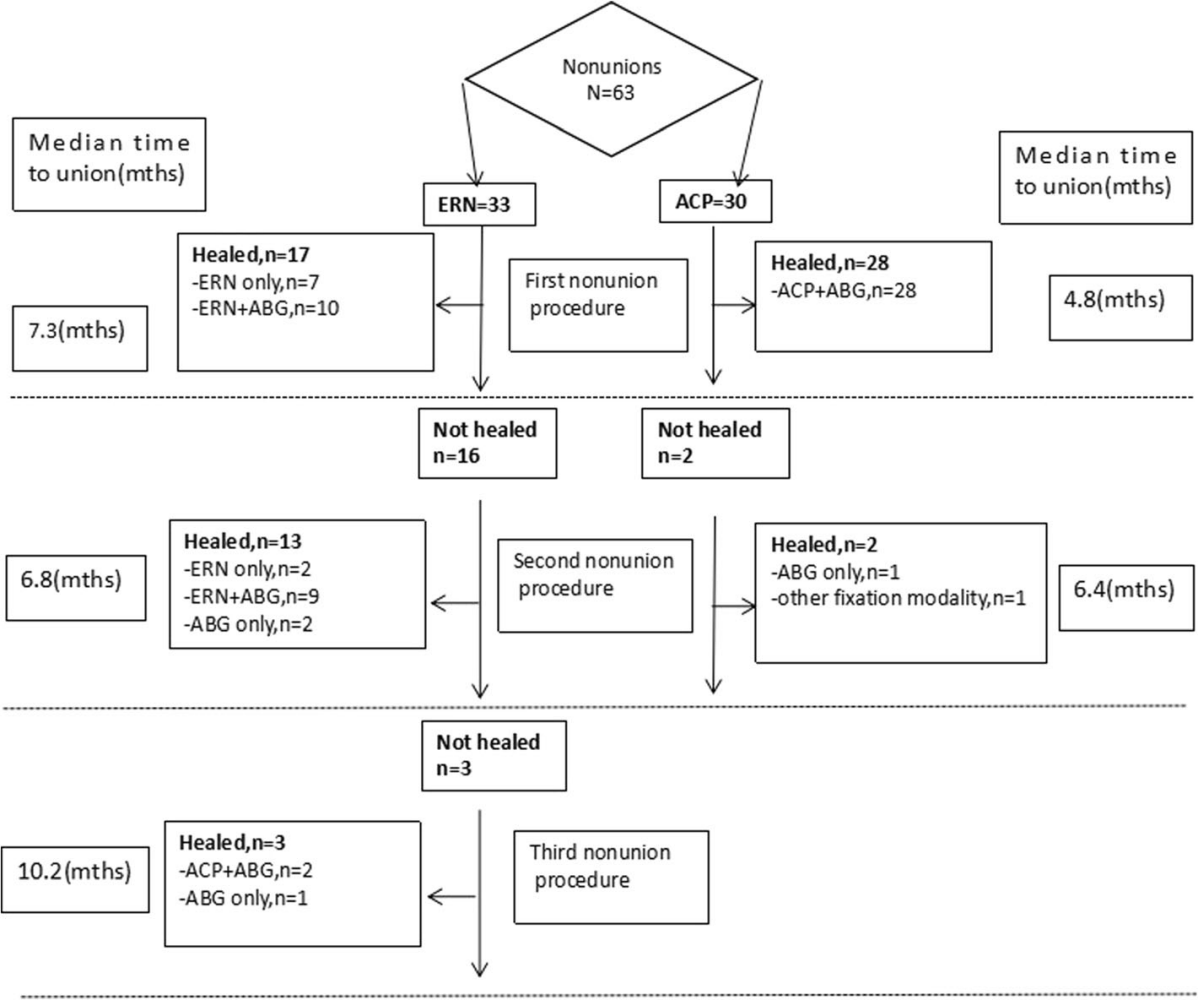
Flow diagram showing outcome of femoral shaft nonunions after failed IMN treated by primary ERN or ACP. IMN, intramedullary nailing; ERN, exchanging reamed nailing; ACP, augmentative compression plating; ABG, autogenous bone grafting

**Fig. 4:**
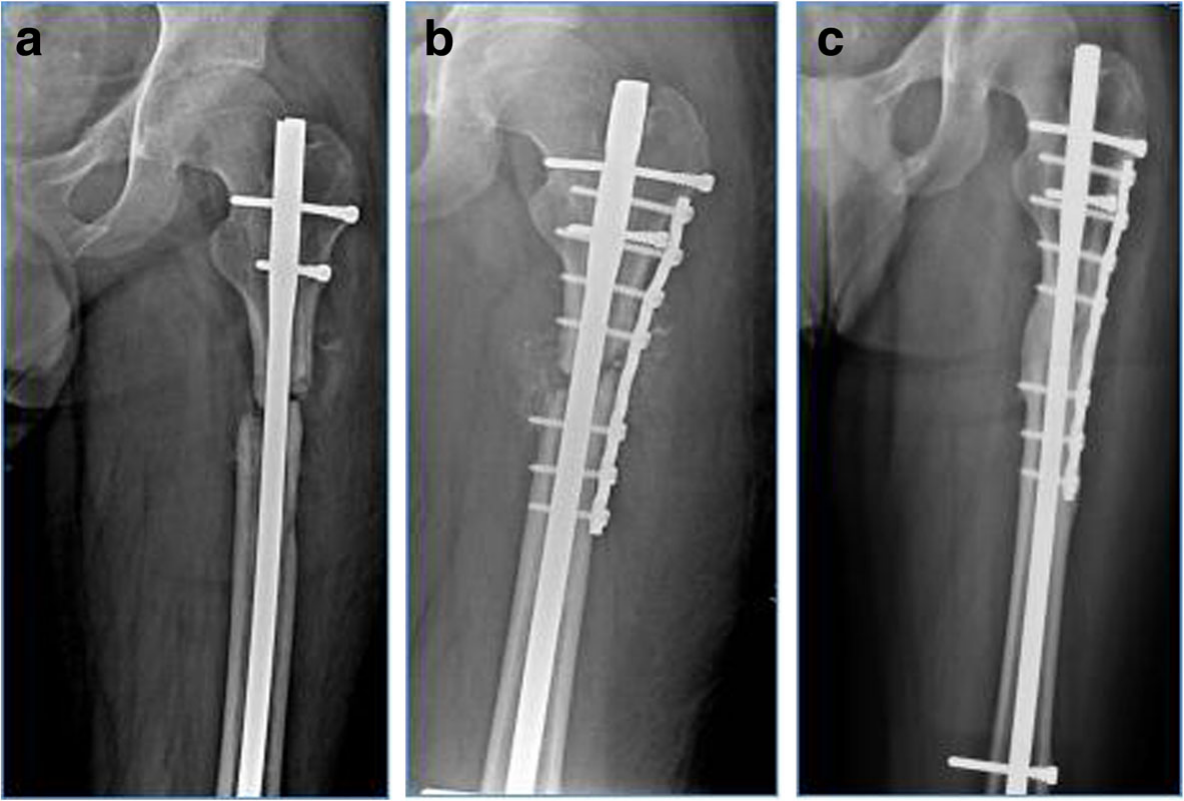
A male patient, aged 39, suffered from femoral shaft supraisthmal fracture following a traffic accident. **a** Anteroposterior X-ray showed femoral shaft nonunion 9 months after treated with antegrade IMN. **b** Instant radiography after primary revision by ACP associated with ABG procedure. **c** Complete bone healing was observed 4 months after revision surgery.ABG, autogenous bone grafting; ACP, augmentative compression plating; IMN, intramedullary nailing

The following factors were associated with the occurrence of re-nonunion in the univariate analysis inclusive of smoking (OR 0.21; *p* = 0.0361), BMI (OR 0.25; *p* = 0.0213), site of nonunion (OR 0.310; *p* = 0.0110), bone defect (OR 0.47; *p* = 0.0232), primary revision method (OR 2.16; *p* = 0.0031), and adjuvant ABG procedure (OR 1.91; *p* = 0.0274). Multiple logistic regression analysis identified primary revision method (OR 1.03; *p* = 0.0021) and adjuvant ABG procedure (OR 1.02; *p* = 0.0030) as two independent risk factors in the development of re-nonunion after primary revision for femoral shaft nonunion subsequent to failed IMN. More specific, the higher nonunion rate was observed in patients revised primarily with ACP compared with those with ERN (Figs. 3 and 4). Likewise, it also was found in patients with adjuvant ABG procedure compared with those without it in primary revision. The statistical results are summarized in Table 3.

**Table 3:** Univariate and multivariable odds ratio of risk factors for the prediction of re-nonunion

Variable	*N*	Comparison	Univariate analyses			Multivariate analyses		
			OR		*p* value	OR		*p* value
			Estimate	95% CI		Estimate	95% CI	
Sex	63	Female vs. male	3.197	0.92, 11.07	0.067	2.801	0.68, 11.49	0.153
Age (years)	63	< 25 vs.25~ 45 vs.> 45	0.816	0.24, 1.41	0.825	0.636	0.30, 2.17	0.589
Smoking	63	Yes vs. no	0.214	0.02, 1.34	0.036*	0.372	0.09, 1.70	0.419
Alcohol abuse	63	Yes vs. no	0.762	0.21, 1.55	0.366	0.791	0.18, 2.02	0.602
BMI (kg/m^2^)	63	< 30 vs.≥ 30	0.253	0.10, 1.49	0.021*	0.376	0.17, 1.74	0.314
Cause of injury	63	Traffic injury vs. falling vs. crushing	2.031	0.86, 4.37	0.102	2.310	0.86, 5.12	0.099
Fracture type	63	Closed vs. open	1.179	0.54, 2.49	0.692	0.820	0.36, 1.89	0.618
Type of IMN	63	Antegrade vs. retrograde	2.6974	0.96, 9.81	0.148	2.315	0.67, 8.19	0.178
Use of IMN locking screws	63	Dynamic vs. static	0.608	0.26, 1.38	0.237	0.826	0.34, 2.18	0.682
Site of nonunion	63	Isthmal vs. non-isthmal	0.310	0.07, 1.52	0.011*	0.403	0.11, 1.81	0.277
Primary nonunion time (years)	63	< 1.5 vs.≥ 1.5	0.918	0.28, 3.20	0.914	0.719	0.23, 2.71	0.616
Pathological type of nonunion	63	Atrophic vs. hypertrophic	0.517	0.28, 1.19	0.146	1.629	0.28, 3.26	0.701
Bone defect (mm)	63	< 5 vs. ≥ 5	0.472	0.12, 2.58	0.023*	0.528	0.18, 2.62	0.302
Primary revision method	63	ERN vs. ACP	2.125	0.09, 1.16	0.003*	1.027	0.03, 1.54	0.002*
Adjuvant ABG procedure	63	Yes vs. no	1.912	0.06, 1.08	0.027*	1.024	0.01, 1.36	0.003*

*N* total number of nonunions, *BMI* body mass index, *IMN* intramedullary nailing, *ERN* exchanging reamed nailing, *ACP* augmentative compression plating, *ABG* autogenous bone grafting, *CI* confidence interval, *OR* odds ratio

*P<0.05

**Fig. 5:**
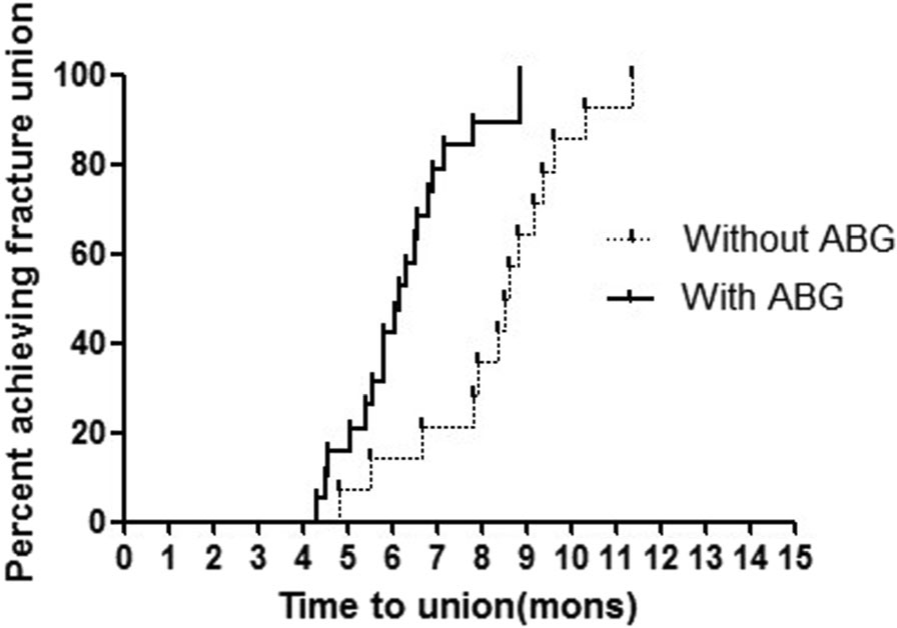
Kaplan-Meier curve of achieving union after primary revision with or without adjuvant ABG. This graph shows time of union in patients with adjuvant ABG procedures was shorter than that without adjuvant ABG procedure. ABG, autogenous bone grafting; IMN, intramedullary nailing; ERN, exchanging reamed nailing; ACP, augmentative compression plating

**Fig. 6:**
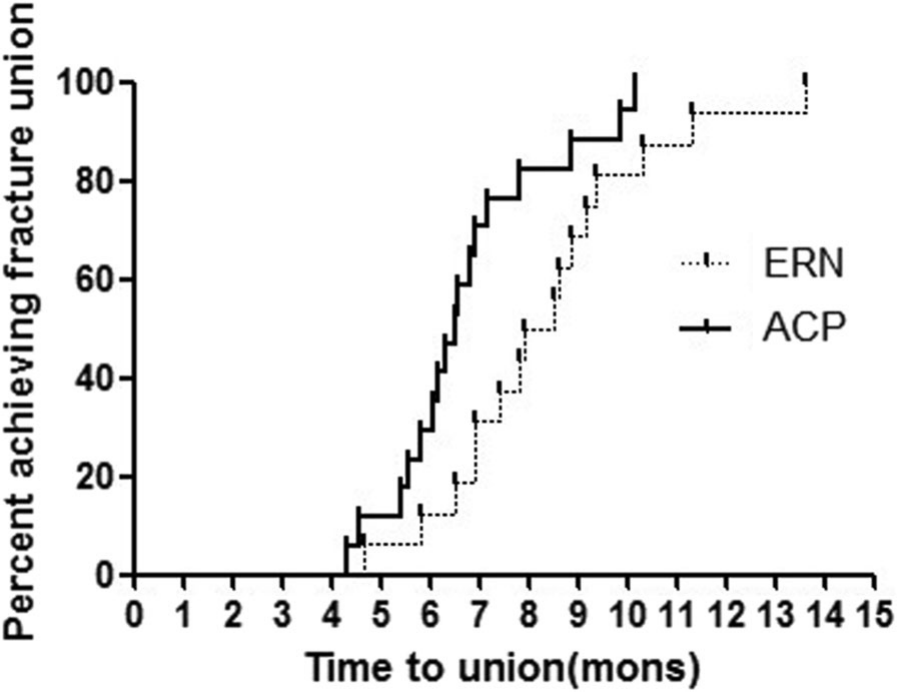
Kaplan-Meier curve of achieving union following primary ERN or ACP surgery. This graph shows time of union in patients following primary ACP surgery was shorter than that following primary ERN surgery. IMN, intramedullary nailing; ERN, exchanging reamed nailing; ACP, augmentative compression plating

Of all patients who undertook primary revision with ERN, 16 (48.5%) patients developed the second nonunion of whom 2 (6.1%) patients obtained the bone union again after second revision with only ABG procedure (Fig. 3). The third nonunion happened in three (9.1%) patients, of whom two (6.1%) patients were converted to the third revision with ACP associated with ABG procedure. Kaplan-Meier curves for the time to union are given in Figs. 5 and 6 with respect to primary revision method (ERN or ACP) and adjuvant ABG procedure (with or without). Bone union was achieved successfully for 17 (51.5%) cases of nonunions with primary revision by ERN and 28 (93.3%) cases by ACP respectively. The median time to union was 10.3 months (IQR 5.9 to 19 months) following primary revision with ERN and 7.6 months (IQR 5.3 to 9.8 months) following ACP for femoral shaft nonunion subsequent to failed IMN; there was a significant difference between two primary revision methods (log-rank, *p* = 0.006). Furthermore, in the primary revision, bone union completed successfully in 34 (87.2%) cases of nonunions with adjuvant ABG procedure and in 11 (45.8%) cases without it respectively. The median time to union was 11.6 months (IQR 6.9 to 19 months) in the absence of ABG and 7.8 months (IQR 5.3 to 9.6 months) in the presence of ABG during primary revisions; this difference was also statistically significant (log-rank, *p* = 0.009).

## Discussion

Despite advances in surgical technique, fracture fixation alternatives, and adjuncts to healing, femoral shaft nonunion after failed IMN continues to be a significant clinical problem as a treatment dilemma for orthopedists [8–10]. Further investigations might allow clinicians to determine whether there are any management strategies that would potentially reduce the risk of specific adverse outcomes and allow patients to maximize their recovery in the most expeditious manner.

The re-nonunion rate of 18/63 (28.6%) was shown in the present study following primary revision with either ERN or ACP for femoral shaft nonunion after failed IMN. Smoking, BMI, site of nonunion, bone defect, primary revision method, and adjuvant ABG procedure were statistically significant risk factors for the occurrence of re-nonunion after primary revision surgery. Two independent risk factors inclusive of primary revision with ERN and no adjuvant ABG procedure increased the median time to union by approximately 3 and 4 months respectively, which raised the chance of requiring a second, even third procedure to achieve bony union. It was indicated that the choice of primary revision with ERN and no adjuvant ABG may be associated to negative outcome after primary revision for femoral shaft nonunion subsequent to failed IMN in our present study.

The healing process of the fracture is very complicated during which there are many affecting factors. Excessive tobacco use can inhibit the osseous formation and mineralization, leading to lower mechanical stability and increasing the risk of nonunions [11]. In addition, it has been found that inhaled carbon monoxide and nicotine in tobacco use can delay the formation of bone collagen by 30~ 40 min and may have an adverse effect on fracture healing. Previous studies have demonstrated a greater union rate in nonsmokers (84%) compared with smokers (58%) [12, 13]. In a review by McKee et al., it was demonstrated that smoking was significantly associated with the development of nonunion (*P* = 0.031) [13]. In our study, smoking was included as a host factor if the patient had a habit of ten or more cigarettes per day; however, we recorded the details if patients reported smoking less frequently. The univariate analysis result showed smoking (OR 0.21; *p* = 0.0361) was associated with development of re-nonunion, which may result from the decrease of bone collagen formation, the increase of bone resorption at the end of fracture, and the prolongation of bone healing time in smokers. A recent study by Westgeest reported that obese patients (BMI ≥ 30 kg/m^2^) have the higher incidence of fatty liver, hyperlipidemia, diabetes, hypertension, coronary heart disease, and pulmonary heart disease, which is about 23 to 40% [12]. In this univariate regression analysis, BMI ≥ 30 kg/m^2^ correlated with the occurrence of re-nonunion (OR = 0.25; *p* = 0.0213). The reasons may include metabolic disturbance resulting from abnormal liver function, required greater violence and severer soft tissue injury in obese patients, excessive dissection of periosteum, larger adverse stress on the fracture end after surgery, and occurrence of infection for poor local immunity. Additionally, the development of nonunion may result from the presence of a gap which is either because of traumatic bone loss or improper surgical treatments such as excessive distraction of fracture ends and bony debridement etc. [6]. In this univariate regression analysis, bone defect with 5 mm or more was associated with the occurrence of re-nonunion (OR 0.47; *p* = 0.0232). In fact, the magnitude of this defect is variable and depends on injury characteristics as well as the physiological function of the patient. When bony bridging can not occur, further intervention should be required to manage the resulting condition of bone defect.

As previously mentioned, the management of a femoral nonunion after failed IMN is challenging. At present, there are a considerable amount of revision methods for femoral shaft nonunion after failed IMN, mainly including ERN and ACP surgery. However, there is no consensus in the literature with regard to their surgical indications [14]. Recently, many studies have questioned the effectiveness of ERN, and its reliability has been suggested to be re-evaluated according to different anatomic sites for femoral shaft nonunion after failed IMN [15–17]. Traditionally, the femoral shaft is divided into upper 1/3, middle 1/3, and lower 1/3, whose limitation lies in having no taken the anatomic variation among individuals into account. We applied the classification method described by Park [9], and femoral shaft was divided into isthmal and non-isthmal (supraisthmal and infraisthmal), giving full consideration to the existence of anatomical features as femoral isthmus and metaphysial flare whose advantage is having less interference from individual anatomic variations. Some studies have shown that ERN had higher failure rates when applied in the treatment of non-isthmal femoral nonunions after failed IMN [18].

However, it also has been reported that ACP associated with ABG procedure could obtain the union rate of 100% for non-isthmal femoral nonunions. Though the univariate regression analysis had shown site of nonunion was associated with the occurrence of re-nonunion (OR 0.310; *p* = 0.0110), this was not confirmed by multivariate regression analysis in our study. All above outcomes suggested the nonunion site had little correlation with the occurrence of re-nonunion [9, 17].

A systematic review analyzed the outcome of revisions with either ERN or ACP for femoral nonunions after failed IMN. The results showed that bone union was seen in 73% (251/343) of patients of 11 inclusive studies with primary revision by ERN at an average of 7 months and 96% (118/122) of patients of 5 inclusive studies with primary revision by ACP at an average of 6 months respectively [8, 19–29]. Based on reports in the literature [9, 28, 29], failure of ERN has been noted in nonunions with severe comminuted fracture, large segmental defects, and metaphysial-diaphysial nonunions. The main mechanical problem in failed IMN seems to be rotational instability. Especially for non-isthmal femoral nonunion, ERN may not provide enough mechanical stability while ACP should be preferred. In a cadaveric fracture model study by Park et al., the augmentive plate group had a 3.3-fold increase in torsional stiffness and 2.6-fold increase in bending stiffness, compared with interlocking IMN group [30]. Therefore, when making surgical decisions and recommendations to patients, the possible failure of the ERN must be considered. In our previous study, it had been shown that the higher rate of bone union and shorter time to union were achieved in primary revision with ACP than in that with ERN for femoral shaft nonunion after failed IMN. Especially for non-isthmal femoral shaft nonunions or isthmal nonunions with larger bone defect, primary revision with ACP could bring more advantages to patients than that with ERN [31–33]. In present study, the univariate and multivariate analysis both revealed primary revision method could correlate with the occurrence of re-nonunion. More specific, the higher rate of re-nonunion was observed in patients revised primarily with ERN (48.5%) compared to those with ACP (6.7%). Furthermore, the median time to union was 10.3 months (IQR 5.9 to 19 months) following primary revision with ERN and 7.6 months (IQR 5.3 to 9.8 months) following ACP for femoral shaft nonunion subsequent to failed IMN respectively; there was a significant difference (log-rank, *p* = 0.006) between two primary revisions. It was suggested that reasonable selection of indications for primary revision and individually surgical management could effectively avoid the occurrence of re-nonunion in the treatment of femoral shaft nonunion after failed IMN.

However, primary revision method was not the only independent risk factor associated with developing re-nonunion. In a retrospective review by Weresh et al. [27], it was reported that a significant number of patients (47%) required adjuvant ABG procedure to achieve the bone union if applied revision by ERN for femoral shaft nonunion after failed IMN. A more recent study by Konda et al. [34] had shown that 47% of patients with nonunion required an open ABG to achieve osseous union, and ABG from iliac crest could provide for excellent outcomes when used for management of the nonunion. Our study also found that adjuvant ABG procedure was significantly associated with successful achievement of bony healing (OR 1.02; *p* = 0.0030) while having the higher rate of re-nonunion (72.2%) following no adjuvant ABG procedure in primary revision. Multiple logistic regression analysis in our present study further identified adjuvant ABG procedure as the other independent risk factor for re-nonunion after primary revision in the treatment of femoral shaft nonunion subsequent to failed IMN. Though adjuvant ABG procedure is more frequently applied along with ACP technique nowadays, it is suggested that open adjuvant ABG procedure should be considered whichever revision technique is primarily attempted to perform in the treatment of femoral shaft nonunion after failed IMN.

To the best of our knowledge, this is the largest cohort of femoral shaft nonunion after failed IMN revised primarily by either ERN or ACP. All patients were collected from a single institution, the Affiliated Hospital of Yangzhou University, which has undertaken ERN or ACP technique for femoral shaft nonunion after failed IMN for over 15 years. Undeniably, several limitations do exist in our study. Firstly, its drawback was lack of a standardized prognostic tool to estimate delayed healing and nonunion as Audige et al. have suggested [35]. Secondly, as a retrospective study, the omitting patients (e.g., those who died or were lost in follow-up) from the analysis can introduce a selection bias. In addition, the number of patents in the re-nonunion group was relatively small. Therefore, caution is needed before drawing conclusions and generalizing our findings to other subjects. Finally, we also are aware that not all potential variables were investigated such as Gustilo type, American Society of Anesthesiologists (ASA) score, and use of NSAIDs (yes or no) etc. Therefore, the research design needs to be improved further in the future study.

## Conclusions

Re-nonunion complication places a cost burden on total healthcare expenditure. Better understanding of the epidemiology and pathogenesis of re-nonunion is essential because it is more important to prevent this complication than to treat it.

The purpose of this study was to evaluate risk factors in the development of re-nonunion after primary revision with either ERN or ACP for femoral shaft nonunion subsequent to failed IMN. Multivariable regression analysis revealed that primary revision method and adjuvant ABG procedures were closely related with the occurrence of re-nonunion. These outcomes have suggested that it is possible, by strictly selecting primary revision method and adjuvant ABG procedures, to reduce the occurrence of re-nonunion in the treatment of femoral shaft nonunion after failed IMN.

## Abbreviations

ABG: Autogenous bone grafting
ACP: Augmentative compression plating
AP: Anteroposterior
BMI: Body mass index
ERN: Exchanging reamed nailing
GEE: Generalized estimating equation
IMN: Intramedullary nailing
